# Sex-specific effects of parasites on telomere dynamics in a short-lived passerine—the blue tit

**DOI:** 10.1007/s00114-019-1601-5

**Published:** 2019-01-30

**Authors:** Joanna Sudyka, Edyta Podmokła, Szymon M. Drobniak, Anna Dubiec, Aneta Arct, Lars Gustafsson, Mariusz Cichoń

**Affiliations:** 1 Centre of New Technologies, University of Warsaw, Banacha 2c, 02-097 Warszawa, Poland; 2 Institute of Environmental Sciences, Jagiellonian University, Gronostajowa 7, 30-387 Kraków, Poland; 3 Museum and Institute of Zoology, Polish Academy of Sciences, ul. Wilcza 64, 00-679 Warszawa, Poland; 4 Department of Ecology and Genetics, Evolutionary Biology Centre, Uppsala University, Norbyvägen 18 D, SE-752 36 Uppsala, Sweden

**Keywords:** Avian malaria, Bird, Chronic infection, Parasitaemia, Random regression

## Abstract

Parasitic infections potentially drive host’s life-histories since they can have detrimental effects on host’s fitness. Telomere dynamics is a candidate mechanism to underlie life-history trade-offs and as such may correlate with observed fitness reduction in infected animals. We examined the relationship of chronic infection with two genera of haemosporidians causing avian malaria and malaria-like disease with host’s telomere length (TL) in a longitudinal study of free-ranging blue tits. The observed overall infection prevalence was 80% and increased with age, constituting a potentially serious selective pressure in our population. We found longer telomeres in individuals infected with a parasite causing lesser blood pathologies i.e. *Haemoproteus* compared to *Plasmodium* genus, but this only held true among males. Female TL was independent of the infection type. Our results indicate that parasitic infections could bring about other types of costs to females than to males with respect to TL. Additionally, we detected linear telomere loss with age, however a random regression analysis did not confirm significant heterogeneity in TL of first breeders and telomere shortening rates in further life.

**Electronic supplementary material:**

The online version of this article (10.1007/s00114-019-1601-5) contains supplementary material, which is available to authorized users.

## Introduction

Parasitic infections have been reported to impair survival in a number of taxa (Valkiūnas 2005; Kilpatrick et al. 2010; Murray et al. 2010; Cooper et al. 2012). Detrimental action of parasites is often delayed, resulting in prolonged coexistence of parasite and host. This fact motivates a query for the proximate mechanism mediating long-term consequences (reduced survival) in animals that were able to withstand acute infection stage. In the light of current knowledge, two main mechanisms could be proposed: behavioural (sick animals often become inefficient in foraging, avoiding predation or defending territory [Owen-Ashley and Wingfield 2007], during both acute and chronic phase of infection) and physiological. In the latter case, resources available to the host can be depleted by the parasite directly and indirectly through activation of the immune system (Hasselquist and Nilsson 2012). Consequently, a trade-off in host’s resource allocation is being compromised by the presence of a parasite, potentially resulting in impaired self-maintenance (Sheldon and Verhulst 1996).

One potential mechanism that could elucidate reduced survival of infected animals is telomere dynamics. Telomeres, non-coding DNA repeats, capping ends of chromosomes, are perceived as a biomarker of viability and quality (Bauch et al. 2013; Boonekamp et al. 2013). Telomeres shorten with each cellular division due to incomplete replication on lagging DNA strand, and as such are associated with the ageing process (Monaghan and Haussmann 2006). Indeed, telomere shortening with increasing age has been confirmed not only *in vitro* in human fibroblasts (Allsopp et al. 1992), but also across multiple free-living and laboratory animal populations (Dantzer and Fletcher 2015; but see Hoelzl et al. 2016; Rollings et al. 2017; Ujvari et al. 2017) and the rate of erosion may indicate cumulative effects of various stressors (Bauch et al. 2014). While the impact of catch-up growth, sibling competition, reproduction or diet composition has been increasingly explored (Geiger et al. 2012; Boonekamp et al. 2014; Sudyka et al. 2014; Noguera et al. 2015), the association between parasitic infection and telomere dynamics is not well understood yet. To our knowledge, apart from observational studies on negative effects of disease on telomere length (TL) in humans (Kong et al. 2013) and mice (Ilmonen et al. 2008), very few studies considered the direct relationship between parasitic infections and telomere dynamics so far. Asghar et al. (2015) showed that great reed warblers infected with avian malaria lived shorter and their telomeres eroded at a faster rate while compared to birds being free of infection. This study suggests that disease-induced negative effects on survival may be mediated through telomere degradation. Other studies also report negative effect of parasitic infections on TL in siskins and tawny owls (Asghar et al. 2016; Karell et al. 2017), however some recent studies failed to show such relationship (Slowinski 2017; Stauffer et al. 2017). This inspires further query on assessing telomere dynamics in various populations in response to parasite-induced diseases.

Avian malaria and malaria-like disease, caused by vector-borne haemosporidian parasites from genera *Plasmodium* and *Haemoproteus*, are among the most common diseases in bird populations (Atkinson and van Riper 1991; Scheuerlein and Ricklefs 2004). The infection becomes chronic in individuals surviving the initial acute phase of the disease (Valkiūnas 2005). While the life cycle of both parasite genera involves development in blood cells, *Haemoproteus* is expected to cause less damage to blood cells. Merogony (asexual reproduction) of *Haemoproteus* occurs in other tissues, whereas *Plasmodium* multiplies also in red blood cells leading to their disintegration (Valkiūnas 2005; Valkiūnas and Iezhova 2017). Blood pathologies induced by parasitaemia are expected to increase demand for new blood cells (Schoenle et al. 2017), which can be attained through higher number of cellular divisions, possibly leading to pronounced telomere erosion. Due to the differences in life cycles between the haemosporidian parasites, lesser impact of *Haemoproteus* on blood TL is expected.

Here, we aim to determine the relationship between infection with blood parasites (from genus *Plasmodium* and *Haemoproteus*) and telomere length (TL) in a wild population of a small, short-lived passerine, the blue tit (*Cyanistes caeruleus*). To this end, we examined if the presence of blood parasites correlates with TL of host’s red blood cells. We expected to (i) detect shorter telomeres among infected individuals while compared to uninfected ones and (ii) find differences in TL among hosts infected with parasite genera with varying life cycles, as the action of each parasite is expected to have diverse direct impact on red blood cells. A longitudinal setup of our study allowed to test if (iii) red blood cell telomeres become shorter with chronological age of an individual and (iv) components of telomere dynamics: first breeders’ TL and TL erosion rate vary among individuals.

## Materials and methods

### Study object

The study was performed in a wild population of nest-box breeding blue tits inhabiting the southern part of the Baltic island of Gotland, Sweden (see Przybylo et al. (2000) for detailed study site description). This population provides a convenient framework for longitudinal studies of age-related parameters due to relatively high survival rate and recapture rate (40% and 41% respectively, (Podmokła et al. 2017)). We have continuously monitored this population since 2002, and for the present analysis we considered data from breeding seasons 2008–2015.

The blue tit is a small passerine (~ 12 g) with a maximum longevity of 12 years recorded in the wild (EURING database). The median lifespan of birds that were observed in our population as adults is 2 years, and maximum lifespan recorded in the study period is at least 6 years. Birds seem to contract infection locally (adult blue tits in this part of Europe rarely migrate (Smith and Nilsson 1987), and even if they do, their wintering grounds are devoid of active malaria vectors), during the activity period of their vectors (May–October): mosquitoes (Culicidae) in the case of *Plasmodium* and biting midges (Ceratopogonidae) and louse flies (Hippoboscidae) in the case of *Haemoproteus* (Valkiūnas 2005). Since the prepatent period of infection usually lasts several weeks (Zehtindjiev et al. 2008; Asghar et al. 2016) and seasonal prevalence of parasites is bimodal with the most pronounced peak in autumn (Cosgrove et al. 2008), the infections that we observe (all samplings took place in May–June) were generally chronic, i.e., had to be contracted at least a few months before the sampling.

Adults were caught while feeding nestlings, 10–14 days after hatching of their young either with mist nets set in the vicinity of nest-boxes or self-releasing traps installed inside nest-boxes. All individuals were ringed, blood sampled, and then immediately released. Birds were aged on the basis of a distinctive moult limit visible between the great and the primary wing coverts (Svensson 1992), and their age was assured on the basis of ringing data. Sex was determined based on the presence (female) or absence (male) of a brood patch. Considering the primary importance of longitudinal sampling in TL analyses (Nussey et al. 2008), we chose a subset of birds that were caught at least twice and the first capture took place in their first breeding season (i.e. 1-year-old birds) to standardise age-related effects across all individuals. Such individuals, surviving beyond their first reproductive season, are presumably of better quality than the birds dying earlier. However, in our population, we did not find any effect of infection status (Podmokła et al. 2017) or TL dynamics (Sudyka et al. 2014) on survival, therefore we do not expect our results to be prone to the potential quality-dependent bias in the studied parameters.

According to our database, 160 individuals were captured at least twice. From the analyses we had to exclude birds that underwent an experimental brood size enlargement in their first breeding season, because we have demonstrated that this manipulation alters TL in the study population (Sudyka et al. 2014). For some individuals a blood sample was not available, therefore our final dataset comprised 246 samples belonging to 112 individuals (up to four samples per individual collected in yearly intervals in 54 males and 58 females).

### Host’s telomere length analysis

Immediately after sampling, whole blood was placed in 96% ethanol until extracted with the Blood Mini kit (A&A Biotechnology, Gdynia, Poland; modified for overnight incubation in 37 °C). The DNA concentration and purity was measured with a NanoDrop 1000 Spectrophotometer (Thermo Fisher Scientific, Waltham, MA, USA) and the integrity of each sample was confirmed by an electrophoresis on a 1% agarose gel.

TL was assessed by the real-time quantitative PCR assay adapted for birds (Criscuolo et al. 2009). We used relative TL, expressed as the ratio (*T*/*S*) of a telomere copy number (*T*) and a single control gene copy number (*S*, which was GAPDH, (Cawthon 2002)). We used the primers designed for zebra finch but these have been previously validated for the blue tit by us (see Sudyka et al. 2014, 2016). To generate a standard curve for amplification efficiency, each plate included serial twofold dilutions of a reference DNA (mixed DNA of five birds not included in the study) run in duplicate from 10 to 0.31 ng for telomere and from 10 to 0.62 ng for GAPDH. The same DNA was used as the *golden* sample to account for inter-plate variation and run in triplicate on every plate. Mean amplification efficiency and the determination coefficient (*r*^2^) of the standard curve were 96% (range 82–109%) and 0.985 (range 0.963–0.990) for GAPDH and 86% (range 77–103%) and 0.974 (range 0.949–0.993) for telomeres respectively. Mean intra-plate SD for the *Ct* values (repeatability as defined by the MIQE guidelines (Bustin et al. 2009)) was 0.23 (coefficient of variation (100 × SD/mean value), CV = 2.00%) for telomeres and 0.12 (CV = 0.48%) for GAPDH, whereas inter-plate CV (reproducibility as defined by the MIQE guidelines) and SD calculated on the golden sample’s *T*/*S* ratios were 17% and 0.09 respectively. All samples from one individual were assayed on the same plate to avoid between plate variations within individual, whereas sexes were evenly distributed on a plate. If the variation between technical replicates (*Ct* SD) exceeded 0.5, all samples for each individual were measured again. In total, we ran 21 plates. For further details on primers, reagent concentration, reaction setup and *T*/*S* ratio calculation please refer to Sudyka et al. (2014). From the analyses of TL, we had to exclude one female due to DNA degradation in the second sample. As a result for TL, we analysed 244 samples from 111 individuals (57 females, 54 males). Repeatability for TL values within individual (calculated with rptR package (Stoffel et al. 2017)) was *R* = 0.223 ± 0.081, CI = 0.056–0.376, *P* = 0.004.

### Malaria status, lineages and infection intensity analyses

To obtain malaria status, the DNA extracted for TL analysis was screened for the presence of blood parasites (genera *Haemoproteus* and *Plasmodium*) by amplifying a 478-bp fragment of the mitochondrial cyt *b* gene, using a nested polymerase chain reaction (Waldenström et al. 2004). After confirming the presence of a parasite, we assessed its lineage by sequencing purified PCR products and aligning DNA sequences with the MalAvi database (Bensch et al. 2009). Intensity of infection (parasite DNA copy number) was then quantified via qPCR, comparing a focal sample with standard curves created using full-length cyt *b* PCR products from *P. circumflexum* and *H. majoris* (as described in (Knowles et al. 2011). (For more details on primers, reaction setups, reagent concentrations and instruments, please see Podmokła et al. (2014a, b)).

### Statistical analyses

To study inter-individual longitudinal variation in TL changes according to malaria infection (parasite-genus wise), we employed random regression analysis, a type of a linear mixed model allowing to study inter-individual variation in intercepts (first breeder TL) and slopes (TL loss with advancing age). To this end, we modelled TL (log-transformed for normality) as a response variable and fitted age (in years, defined as a fixed continuous covariate), sex and infection type as fixed explanatory variables. Infection type was categorised into three levels: none, *Plasmodium* and *Haemoproteus*; mixed infections with both genera (7 cases) were included in *Plasmodium* category because more detrimental effects are expected to arise from the action of this parasite (Valkiūnas 2005); however, we also repeated the analysis excluding mixed infections and the results remained qualitatively the same. We also introduced individual identity to account for the same individual being entered more than once in the analyses, nest identity (some birds attended the same nest), year of data collection and plate id from TL analyses (to account for among-plate variance) as random variables. To test for non-linearity of age effect in initial models, we introduced polynominal (quadratic) age term, but it turned out to be insignificant, so was culled from the final model. We began with full models, and then we culled non-significant (*P* > 0.05) interaction terms. Additionally, to evaluate infection status dynamics (response variable: infected vs uninfected) with individual age (explanatory continuous variable), we performed an analysis accounting for individual id in random effects structure. Analyses were performed in R (v.3.3.1) (R Core Team 2016) using the ASReml-R package (Butler 2009).

#### Data accessibility

Data are available in the online Electronic Supporting Information and in the online data repository figshare (Sudyka et al. 2018).

## Results

### Dynamics of infection prevalence and intensity

Overall, haemosporidian prevalence reached 80% (please see the Electronic Supporting Information (ESI), Table S1A) and in 246 samples, we found 216 cases of infection with various parasite lineages from *Plasmodium* (7 lineages, prevalence 56.5%) and *Haemoproteus* genera (3 lineages, 20.3% prevalence), whereas 2.8% of birds yielded mixed infection with the two genera (Table S1B, ESI). Prevalence (proportion of infected individuals) increased with age (estimate for age ± SE = 0.624 ± 0.239, *F*_1,242_ = 6.847, *P* = 0.009). Among 102 individuals, that were recaptured in the subsequent year, probability of contracting infection was high (0.71), whereas losing infection was less likely (0.12) (Table S2, S3, ESI). As a result, prevalence of infection among 1-year-old birds was 73% and it reached 83% in two-year-olds (Table S2, ESI). Infection with *Haemoproteus* was generally more intensive than with *Plasmodium* (intensity for all present lineages, excluding mixed infection with both genera, Mann-Whitney *U* = 594, *N* = 186, *P* < 0.001).

### Longitudinal telomere dynamics vs avian malaria

**Fig. 1:**
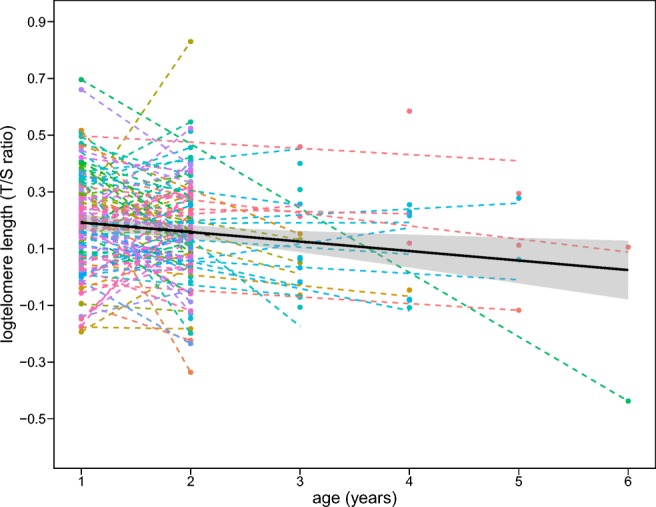
Telomere loss in relation to age in the blue tit. Individual regression lines (*N* = 111 individuals, sampled 2 to 4 times) represented in colours, dots represent sampling events. Overall regression line ± SE (pooled for all individuals) shown in black on raw data

**Fig. 2:**
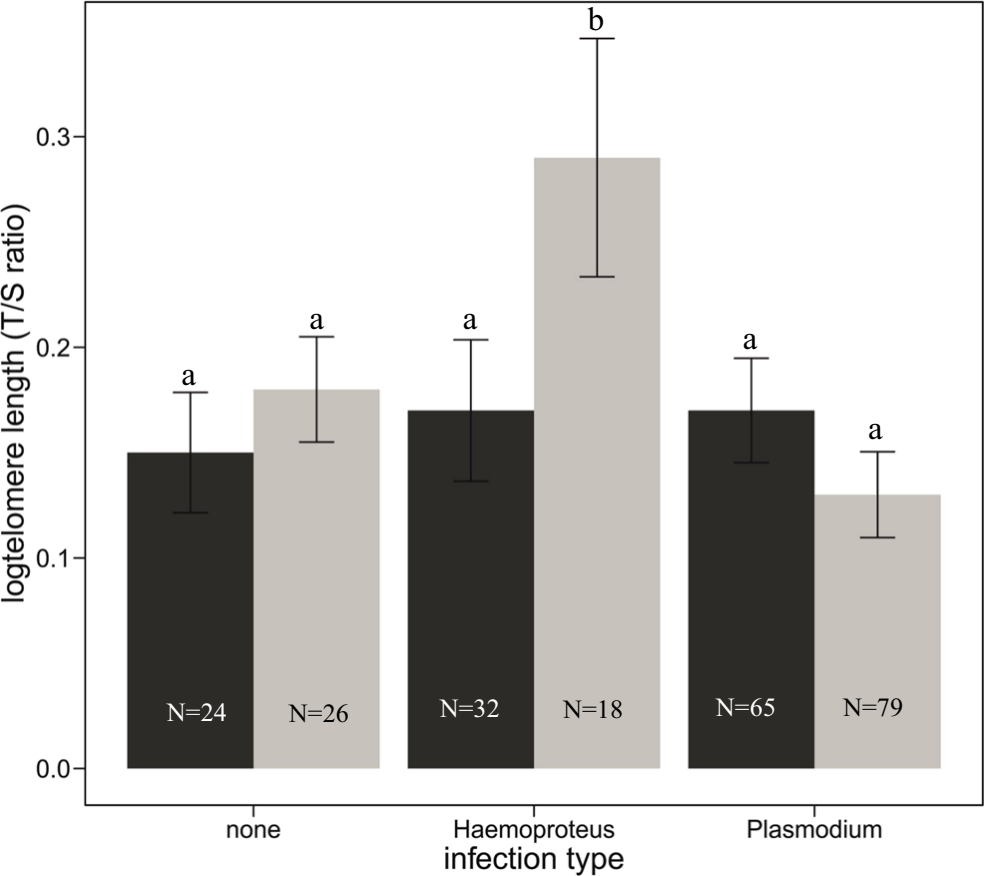
Relative telomere length [the ratio (*T*/*S*) of telomere copy number (*T*) and single control gene copy (*S*) in females (dark grey bars) and males (light grey bars) according to infection type. Raw data ± SE are shown. Differences among groups (non-adjusted *P* values) according to the post hoc pairwise mean difference test with Holm false-positive rate discovery correction

Including a covariance between individual slopes and intercepts did not improve the fit of our model (log(likelihood) of a model including the correlation = 284.13, and of a model without the correlation = 283.99, likelihood-ratio test *P* = 0.597), thus we applied the model without the covariance. Random regression analysis (Table 1) revealed that the variations in intercepts (first breeder TL) and among individual regression slopes (TL attrition rate) were not significant. We found a significant negative effect of chronological age on TL (Table 1, Fig. 1). TL was found to be significantly correlated with malaria infection type; we found longer telomeres in individuals infected with *Haemoproteus* compared to *Plasmodium*, but this only held true among males (Table 1, Fig. 2). Uninfected males showed intermediate levels of TL between the ones infected with the two parasite genera. Infection type was not related to TL among females (Table 1, Fig. 2). Telomere attrition rate (telomere shortening with age) was sex independent (age × sex, *F*_1,100.9_ = 1.869; *P* = 0.175), and more importantly, it was also not related to the type of parasitic infection (age × infection type, *F*_2,166.2_ = 0.579; *P* = 0.562), so these interactions were not included in the final model. We found no association between infection intensity and TL (model description and results in Table S4, ESI).

**Table 1:** General linear mixed model explaining the effects of age, sex, and malaria infection type on telomere length in blue tit individuals (each sampled 2 to 4 times in yearly intervals). Significant fixed effects (*P* < 0.05) and components for random effect structure (Z ratio ≥ 1.96) marked in italics

Fixed effects	Estimates ± SE	Z ratio	df	*F*. con	*P*
Intercept	0.236 ± 0.045	5.292	1, 18.0	67.530	*< 0.001*
Age	− 0.043 ± 0.014	− 3.129	1, 56.7	9.788	*0.003*
Sex	0.143 ± 0.056	2.570	1, 104.2	0.683	0.410
Infection type	0.007 ± 0.040	0.180	2, 220.2	1.860	0.158
Sex × infection type			2, 219.5	3.348	*0.037*
Male × none	− 0.116 ± 0.071	− 1.628			
Male × *Plasmodium*	− 0.163 ± 0.063	− 2.586			
Random effects	Component ± SE	*Z* ratio			
Individual intercepts (first breeder TL)	0.003 ± 0.003	1.114			
Individual slopes (TL loss with age)	0.001 ± 0.001	1.137			
Nest id	0.002 ± 0.007	0.275			
Year	0.004 ± 0.004	1.192			
Plate id	0.006 ± 0.003	*2.056*			
Residual variance	0.018 ± 0.007	2.427

## Discussion

In line with our expectations, infection type affected blood TL. We found that males infected with *Haemoproteus* had longer telomeres than males infected with *Plasmodium*. Such a finding can be readily explained, considering differential life cycles of the two genera. *Haemoproteus* causes less blood pathologies during its developmental cycle (Atkinson and van Riper 1991; Valkiūnas 2005), therefore the demand for new blood cells via telomere shortening cellular divisions is lower.

Conversely to the previous studies in some bird populations (Asghar et al. 2015, 2016; Karell et al. 2017), we did not show that uninfected birds had longer telomeres. Uninfected males showed intermediate TL between the ones infected with *Plasmodium* and *Haemoproteus*. This lack of effect has already been reported in other animals (Slowinski 2017; Stauffer et al. 2017) and adds to the evidence that parasitic infections do not always entail negative consequences on TL. In general, the information on the relationship between telomere dynamics and parasitic infections is still lacking, and comparisons among taxa have to be treated with great caution since telomere dynamics and telomerase activity varies substantially within animal kingdom (Olsson et al. 2018). Alternatively, temporal resolution of our sampling points—we miss the information on autumn prevalence—could be not fine enough to detect the differences (expected especially due to the acute stage of infection (Asghar et al. 2016)). For example, in humans, malaria infection considerably shortened telomeres up to 3 months post infection, but after 1 year, TL was restored (Asghar et al. 2018). It appears that varying results among studies examining the impact of parasitic infections on TL may stem from different phases of infection investigated. Since we demonstrate that *Haemoproteus*-infected males have longer telomeres than the other groups, we would expect that the rate of their telomere loss is lower. However, since we did not detect significant differences in shortening rates and TL as first breeders among individuals (Table 1), telomere dynamics of these males could have been shaped prior to the first sampling. To our knowledge, this is the first study to show diverse relationship of parasite genera varying in life cycles on TL and stresses the importance of accounting for ecological structure (parasite genera with different developmental patterns in this case) while addressing parasite–host interactions. Indeed, it has already been demonstrated that avian malaria can yield multiple effects on their hosts (Lachish et al. 2011), yet exploring early-life telomere dynamics in response to such interactions could be particularly needed.

We show that effect of host interaction with parasite may also be sex-specific. Unlike males, females infected with *Haemoproteus* did not show longer telomeres in the studied population. It is possible that infection with *Haemoproteus*, incurs other types of costs to females than to males. For example, anti-malarial treatment reduced *Haemoproteus* infection intensity and ultimate fitness cost, i.e. survival only in females, whereas males did not benefit from such a manipulation (Martínez-de la Puente et al. 2010). While males are generally more susceptible to parasitic infections, endocrine-immune interactions may cause increased resistance to some parasites in males (Klein 2004).

We demonstrated that TL decreases linearly with advancing individual age in both sexes and parasitic infection type does not alter the general pattern. The number of longitudinal studies in natural populations indicating telomere erosion with age slowly, but persistently increases (Dantzer and Fletcher 2015; Sudyka et al. 2016). However, the causal involvement of telomeres in ageing process still remains an open question (Simons 2015). This discussion is further inspired by recent longitudinal studies indicating no telomere erosion (Ujvari et al. 2017) or even telomere elongation with age (Hoelzl et al. 2016), suggesting that divergent life-histories among taxa may shape lifetime telomere dynamics. A longitudinal approach is particularly useful for disentangling within-individual reactions from between-individual heterogeneity (Nussey et al. 2008). Although several studies show variability in individual TL dynamics (Hall et al. 2004; Bize et al. 2009; Heidinger et al. 2012), here we did not confirm statistical significance of heterogeneity neither in TL among first breeders, nor in TL erosion rates. We were only able to detect larger contribution of first breeders TL, rather than telomere erosion rate, to overall variation in TL (Table 1). However, it is important to note, that the lack of significant differences in individual starting TL and erosion rates, does not denote that all individuals share the same erosion rate or have identical TL as first breeders (the variances are non-zero), but that we failed to statistically detect these differences. Even though the number of individuals that we sampled appears to be sufficient, the total sample size falls below the one recommended for individual heterogeneity modelling (van de Pol 2012). While the number of replicates within an individual are restricted by the blue tit’s lifespan, our result motivates and justifies a query in search for factors contributing to the phenotypic TL variation among individuals in other study systems.

To conclude, the idea that TL dynamics may be shaped by various life’s insults is already well established (Monaghan and Haussmann 2006) and here, we showed that parasitic infections may be one of such TL modulating factors in a wild population of a short-lived vertebrate. For the first time, we demonstrated the contrasting associations between two parasite genera varying in life cycles with blood TL. Consistently with majority of the studies on endotherms, in the blue tit telomeres eroded with age on a within-individual level, and this erosion rate was not significantly variable among individuals. Since our study provides correlational evidence for sex-specific differences in TL in response to different parasite genera, it has to be treated as a prerequisite for further exploration of the topic. For example, it could be fruitful to experimentally examine the impact of varying parasite genera on TL in multiple body tissues in a sex-specific context.

## Electronic supplementary material

ESM 1 (PDF 707 kb)
