# Supplementary material for: Sex-specific effects of parasites on telomere dynamics in a short-lived passerine—the blue tit
Source: Naturwissenschaften. 2019 Jan 30;106(1):6. doi: 10.1007/s00114-019-1601-5 (PMC6353807; doi:10.1007/s00114-019-1601-5)
Supplement: Supplementary file 1 — (PDF 707 kb) [file 114_2019_1601_MOESM1_ESM.pdf]

Electronic Supporting Information

Sex-specific effects of parasites on telomere dynamics in a short-lived passerine- the blue tit

The Science of Nature 2019

Joanna Sudyka[1,2*], Edyta Podmokła[2], Szymon M. Drobniak[2], Anna Dubiec[3], Aneta Arct[2], Lars Gustafsson[4], Mariusz Cichoń[2]

[1] Centre of New Technologies, University of Warsaw, Banacha 2c, 02-097 Warszawa, Poland

[2] Institute of Environmental Sciences, Jagiellonian University, Gronostajowa 7, 30-387 Kraków, Poland

[3] Museum and Institute of Zoology, Polish Academy of Sciences, ul. Wilcza 64, 00-679, Warszawa, Poland

[4] Department of Ecology and Genetics, Evolutionary Biology Centre, Uppsala University, Norbyvägen 18 D, SE-752 36 Uppsala, Sweden

*Corresponding author: joanna.sudyka@cent.uw.edu.pl

Table S1. (A) Prevalence of avian malaria in the blue tit population inhabiting island of Gotland (Sweden) in years 2009-2015 (n = 246 samples from 112 individuals, 54 males, 58 females, caught at least twice, first capture as 1 year olds)

NO OF LINEAGES (GENUS)		*N CASES*	*M*	*F*	*PREVALENCE (%)*
**single infection:**		**176**	**87**	**89**	**71.5**
	Plasmodium	126	69	57	51.2
	Haemoproteus	50	18	32	20.3
**double infection:**		**20**	**10**	**10**	**8.1**
	double Plasmodium	13	7	6	5.3
	double mixed (Plasmodium+Haemoproteus)	7	3	4	2.8
**INFECTED individuals**		**196**	**97**	**99**	**79.7**
**UNINFECTED individuals**		**50**	**26**	**24**	**20.3**

(B) Diversity and frequency of detected malaria lineages in n = 246 screened samples

PARASITE TAXON	***LINEAGE***	***GenBank ACCESSION NO***	***N CASES (incl. double lineages)***	***M***	***F***
Plasmodium circumflexum	pTURDUS1	AF495576	104	59	45
Plasmodium circumflexum	pBT7	AY393793	32	12	20
Plasmodium relictum	pSGS1	AF495571	14	*11**	*3**
Plasmodium polare	pSW2	AF495572	5	3	2
Plasmodium relictum	pGRW11	AY831748	2	1	1
Plasmodium sp.**	pBLUTI02	DQ991072**	1	0	1
Plasmodium sp.**	pDELURB4	EU154346**	1	0	1
**pooled *Plasmodium***			**159**	**86**	**73**
Haemoproteus majoris	hPARUS1	AF254977	54	21	33
Haemoproteus majoris	hPHSIB1	AF495565	2	0	2
Haemoproteus majoris	hWW2	AY831755	1	0	1
**pooled *Haemoproteus***			**57**	***21****	***36****
Plasmodium* + *Haemoproteus			**216**	**107**	**109**

*Significant difference in frequency of parasite morph between sexes according to a simple proportion test.**These lineages have not yet been assigned to any species according to MalAvi database

Table S2. Repeatability of infection status in blue tits caught in two consecutive breeding seasons (as one year olds and two year olds) n = 102 individuals

1 YEAR OLDS		***2 YEAR OLDS***			
		**Not infected (n=17)**	**Infected (n=85):**		
			Plasmodium	*Haemoproteus*	*Double (H+P)*
**Not infected (n=28)**		8	16	3	1
**Infected (n=74):**		9	46	18	1
	Plasmodium	6	40 (30)	4	1
	Haemoproteus	3	4	13 (13)	
	Double (H+P)		2	1	

In parentheses: cases with the same lineage of avian malaria in both seasons

Table S3. Changes in infection status in n = 102 individuals (48 males, 54 females) caught in two subsequent breeding seasons (as one year olds and two year olds)

STATUS DYNAMICS		***N CASES***	***M***	***F***	***FREQUENCY (%)***
**no change in status:**		**73**	**36**	**37**	**71.6**
	remain uninfected	8	5	3	7.8
	remain infected	65	31	34	63.7
**got infected**		**20**	**9**	**11**	**19.6**
**lost infection**		**9**	**3**	**6**	**8.8**

Parasite infection intensity and telomere length

To examine relation of parasite infection intensity with telomere length (TL) we modelled TL (log-transformed for normality) as a response variable in a random regression framework (to study inter-individual variation in slopes and intercepts of TL) and fitted age (in years) and total infection intensity of all parasite lineages present within an individual defined as fixed continuous covariates with sex as a fixed explanatory variable. We also introduced individual identity to account for the same individual being entered more than once in the analyses, nest identity (some birds attended the same nest), year of data collection and plate id from TL analyses (to account for among-plate variance) as random variables. We began with full models, and then we culled non-significant (P > 0.05) interaction terms. Results are shown below, in Table S4.

Table S4. General linear mixed model explaining the effects of age, sex and malaria infection intensity on telomere length in blue tit individuals (each sampled 2 to 4 times in yearly intervals). Significant fixed effects (P < 0.05) and components for random effect structure (Z ratio ≥ 1.96) marked in bold.

Fixed effects	**Estimates ± SE**	**Z ratio**	**df**	**Fcon**	**P**
intercept	0.287 ± 0.050	5.775	1, 8.7	34.060	**0.0003**
age	-0.040 ± 0.015	-2.721	1, 65.4	7.377	**0.008**
sex	-0.009 ± 0.025	-0.350	1, 98.3	0.124	0.726
infection intensity	>-0.001± <0.001	-0.379	1, 213.3	0.147	0.704
Random effects	**Component ± SE**	**Z ratio**			
individual intercepts (first breeder TL)	0.003 ± 0.003	1.074			
individual slopes (TL loss with age)	0.001 ± 0.001	1.260			
nest id	<0.001 ± <0.001	**7.486**			
year	0.010 ± 0.008	1.231			
plate id	0.007 ± 0.003	**2.103**			
residual variance	0.019 ± 0.003	7.486			
